# Effects of alternate day calorie restriction and exercise on cardio-metabolic risk factors in overweight and obese adults: an exploratory randomized controlled study

**DOI:** 10.1186/s12889-018-6009-1

**Published:** 2018-09-15

**Authors:** Minsuk Oh, Sue Kim, Ki-Yong An, Jihee Min, Hyuk In Yang, Junga Lee, Mi Kyung Lee, Dong-Il Kim, Hye-Sun Lee, Ji-Won Lee, Justin Y. Jeon

**Affiliations:** 1 Department of Health and Human Physiology, University of Iowa, Iowa City, IA USA; 2 Department of Family Medicine, Severance Hospital, Yonsei University, College of Medicine, Seoul, South Korea; 3 Faculty of Physical Education and Recreation, University of Alberta, Edmonton, AB Canada; 4 Department of Sport and Leisure Studies, Yonsei University, Seoul, South Korea; 5 Exercise Medicine Center for Cancer and Diabetes Patients, ICONS, Yonsei University, Seoul, South Korea; 6 Graduate School of Sport Science, Kyung Hee University, Yongin-Si, South Korea; 7 Department of Professional Therapy, Graduate School of Professional Therapy, Gachon University, Geonggi-do, South Korea; 8 Department of Biostatistics, Yonsei University, College of Medicine, Seoul, South Korea; 9 Department of Family Medicine, Gangnam Severance Hospital, Yonsei University College of Medicine, Seoul, Republic of Korea

**Keywords:** Obesity, Exercise, Alternate day calorie restriction, Body weight, Insulin resistance

## Abstract

**Background:**

It has been recognized that alternate day calorie restriction (ADCR) or exercise has positive effects on cardio-metabolic risk factors. It is unclear whether the combined effect of ADCR and exercise (aerobic + resistance training) influences risk. We investigated effects of an 8-week ADCR and exercise program (aerobic + resistance training) on cardio-metabolic risk factors in overweight and obese adults.

**Methods:**

This study randomized 45 overweight or obese but healthy adults (F = 26, M = 19; aged about 32 to 40 years) into 4 groups: ADCR (*n* = 13), exercise (*n* = 10), exercise plus ADCR (*n* = 12), and control (n = 10) for 8 weeks. Body composition, blood lipids profile, and insulin resistance were measured. The intention to treat (ITT) method was used to analyze all participants that were randomized.

**Results:**

A total of 35 participants completed the trial (78%). Body weight, body mass index, waist circumference, fat mass and percent body fat were reduced in the exercise plus ADCR group (− 3.3 ± 2.4 kg, *p* < 0.01; − 1.3 ± 1.0 kg/m^2^, *p* < 0.01; − 4.1 ± 3.9 cm, *p* < 0.01; − 2.7 ± 2.0 kg, *p* < 0.01; − 2. 5 ± 2.2%, *p* < 0.01). Insulin, glucose, homeostasis model assessment insulin resistance and triglyceride (− 2.9 ± 4.1 μIU/ml, *p* < 0.05; − 10.9 ± 16.9 mg/dl, *p* < 0.05; − 0.9 ± 1.3, *p* < 0.05; − 43.8 ± 41.9 mg/dl, *p* < 0.01) decreased in the exercise plus ADCR group only.

**Conclusions:**

ADCR and exercise both proved to be beneficial, but the combined intervention was most effective at inducing beneficial changes in body weight, body composition, glucose, insulin, insulin resistance and triglyceride in overweight and obese adults.

**Trial registration:**

ClinicalTrials.gov: NCT03652532, Registered August 28, 2018, ‘retrospectively registered’.

**Electronic supplementary material:**

The online version of this article (10.1186/s12889-018-6009-1) contains supplementary material, which is available to authorized users.

## Background

Increasing energy expenditure and calorie restriction are the primary methods of care for obesity management; therefore, various manners of exercise and diet are continuously being investigated in overweight and obese individuals [1–6]. Alternate day calorie restriction (ADCR) is one of the calorie restriction modes in weight loss and has been shown to improve health-related outcomes [6–9]. Recent clinical studies have investigated the effects of ADCR in humans on significantly reduced body weight and the risk of coronary heart and artery disease [7, 8, 10].

In contrast to other types of diet protocol, ADCR is composed of “feed days” (ad libitum food intake) and “fast days” in repetition, and has been investigated to be a way of reducing body weight and improving health-related factors in adults [6–9, 11–14]. After 12 weeks of ADCR intervention (consumed 25% of baseline energy requirements in the calorie restriction days) in normal and overweight participants, significant reductions in body weight and fat mass were observed, and cardiovascular health, represented by blood lipids such as triglyceride (TG) or low-density lipoprotein cholesterol (LDL-C), was improved [7]. Furthermore, a recent randomized controlled study in health adults aged 18–65 years investigated that the 24 weeks of ADCR intervention significantly improves fat free mass: total mass ratio, compared to control group [15]. However, to date, a very limited number of studies [16, 17] have examined a trial to investigate the novel effects of ADCR with exercise, or compared the effects between calorie restriction and exercise on body composition or blood lipids profile change in overweight and obese adults.

Bhutani et al. [16] conducted a 12-week randomized controlled trial of aerobic exercise and ADCR interventions with obese adults to compare and investigate the effects between the interventions. ADCR and exercise combined group showed greatest changes in body weight, body fat mass, waist circumference (WC), and CHD risk indicators, such as LDL-C and high-density lipoprotein cholesterol (HDL-C), compared to ADCR group or exercise groups, after 12 weeks of intervention. However, Bhutani et al. [16, 17] conducted their study with solely aerobic type exercise and ADCR intervention. Although all intervention groups in the study preserved lean body mass, there was no increase of lean body mass after the intervention. Hence, ADCR and exercise composed of aerobic type and resistance training could maintain lean body mass and in turn may cause more profound positive effects.

Therefore, the purpose of the study is to examine the combined effects of ADCR and exercise, composed of both aerobic and resistance training, on body weight, body composition, blood lipids, and insulin resistance; additionally, to compare the effects of exercise plus ADCR, ADCR only, and exercise only on the same variables in overweight and obese Korean adults. We hypothesized that participants who participated in both interventions (ADCR + exercise) would show the largest reduction in body weight, change in body composition, and insulin resistance after the 8 weeks.

## Methods

### Study design and procedure

The participant recruitment process was repeated two times from March to April and June to July 2014. To compare the effects of ADCR and exercise, all eligible participants were randomly assigned into 4 groups according to the stratification of demographic factors such as BMI, age, and sex: exercise plus ADCR (E- ADCR), ADCR only, exercise only, and control. Permuted-block randomization was used to allocate all participants into 4 groups in a 1:1:1:1 ratio using a computer-generated random number sequence. Additionally, all of the trained exercise specialists who took the measurements were blinded to group allocation. An orientation session was held before the beginning of the intervention to educate the whole research process and introduce the ADCR and exercise protocol for 8 weeks. After the orientation, baseline measurements (body composition, blood samples, and physical fitness test) were conducted, and post intervention measurements were conducted after 8 weeks of intervention. Text messages were sent three times a week, to all participants according to groups, to encourage participation and increase adherence to the 8 weeks of intervention. Daily dietary logs were provided to all participants and they were encouraged to track their dietary behavior for 8 weeks. Participants who were allocated to the control group were encouraged to spend their time as they usually would. During the whole 8 weeks of intervention, all participants were instructed to fill out the dietary log every day. For the exercise groups, exercise logs were provided and participants were instructed to log every session of exercise. The interventions consisted of a total of 24 ‘fast-days’ and 24 exercise sessions. Participants missing more than 8 days of calorie restriction or exercise (≥ 30%) were considered as low-compliance and were excluded, but not from intention to treat (ITT) analyses.

### Study participants

A total of 50 overweight and obese people made contact through advertisements placed on the Yonsei University campus, and Severance Hospital at Seoul, South Korea. The individuals who were interested in the study were screened according to inclusion criteria by telephone screening. The criteria were as follows: aged 18–64 years; body mass index (BMI) over 23.0 kg/m^2^ (overweight or obese, according to Asian-Pacific criteria from World Health Organization expert consultation) [18]; no weight variation > 5 kg during the previous 3 months; no history of gastrointestinal surgery; not second degree obesity (on hypothyroidism medication); not diagnosed with type 1 or 2 diabetes mellitus; aspartate amino-transferase or alanine amino-transferase levels < 200 mg/dl; no pancreatitis or its related disorder; no infectious diseases (i.e., pneumonia, acute enteritis, or urinary infection); no chronic inflammatory diseases (i.e., rheumatoid arthritis, or lupus); no history of cardiovascular related diseases (i.e., acute myocardial infarction, stroke or angina); no history of cancer; no prescribed medication (i.e., hypertension, diabetes mellitus, diuretic, central-nervous system, antidepressant, antibiotics, anti-inflammatory steroid, or cyclosporin); no pregnant or lactating women; no overeating behavior on ordinary days; no more than 30 g of daily alcohol intake; not a night-time or shift-work worker; not having chronic malabsorption syndrome or cholestasis; no contraindications for participating in exercise and physical test. All procedures of the study were approved by the institutional review boards at the Severance Hospital. All participants voluntarily participated in the study, and provided written informed consent prior to participation in the study.

### Measures

Body weight and body composition including percent body fat, muscle, and fat mass were measured by bio-impedance analyzer (Inbody 720, Biospace, Seoul, South Korea) after overnight fasting including water (> 12 h) [19]. Height was measured by electric extensometer (BSM 330, Biospace, Seoul, South Korea). The participants were given light indoor clothing to wear during assessment. BMI was calculated by dividing weight (kg) by the square of height (m). WC was measured by tapeline to the nearest 0.1 cm, at the belly button in standing posture. Blood pressure was measured by electronic manometer (BPBio 320, Biospace, Seoul, South Korea). Heart rate was measured by a Polar heart rate monitor (Polar USA, Inc., NY). All measurements were assessed by trained exercise specialists, and the same specialist measured each measurement. After overnight fasting (> 12 h), blood samples of all participants were collected between 8 am and 9 am. Blood samples were immediately centrifuged for 10 min, and separated serum was stored at − 80 °C. Fasting levels of glucose, TC, TG, HDL-C, and LDL-C were assayed using an ADIVA 1650 (Siemens, Tarrytown, NY, USA), and insulin was assayed with an Elecsys 2010 (Roche, Indianapolis, IN, USA). Insulin resistance was assessed using the homeostasis model assessment (HOMA-IR) index [Insulin (mUl/L) X fasting glucose (mg/dl)/405] [20]. All study variables at post-intervention were measured on Saturday which was a day right after the end of 8th week of intervention.

### Diet intervention

Only participants in the E-ADCR and ADCR groups participated in the 8 week dietary intervention after the education of the calorie restriction. The participants consumed 25% of daily recommend energy intake in 3 days alternately on ‘fast days’ (400–500 kcal), and consumed ad libitum on the remaining 4 days of the week, known as ‘feed days’. On the ‘calorie restriction day’, the participants were instructed to consume food between 12 pm and 2 pm. A composition of daily diet examples was provided in the daily dietary log. All participants of the study were instructed to record their daily dietary intake during the 8 weeks.

### Exercise intervention

Participants in both E- ADCR and exercise group participated in resistance training and aerobic exercise at the research center gym at least 3 times/week for 8 weeks, and exercise logs were provided to the participants. For the first week of intervention, the participants were supervised and exercised one-on-one with a trained exercise specialist, and for the rest of the 7 weeks, participants trained individually. Although it was unsupervised exercise intervention for the 7 weeks, all participants in exercise group were assisted by certified trainers in the research center gym. The exercise logs were monitored every week of the 8 weeks. Participants in E- ADCR group were asked to participate in the exercise on ‘feed days’.

One session of exercise included the following components: 1) 5 min of warm-up; 2) 40 min of resistance training; 3) 20 min of aerobic exercise; 4) 5 min of cool-down. The resistance training was performed using weight training machines, barbells, and dumbbells. The intensity of the resistance training was individualized according to the muscle strength level of each participant, which was assessed by 10 repetition maximum (RM). The training intensity was altered periodically each week: at 1st and 4th week, 70% of 10RM; at 2nd, 5th, and 8th week, 80% of 10RM; at 3rd, and 6th week, 90% of 10RM; and at 7th week, 100% of 10RM. Three sets of 4 exercises (Day 1: Chest Press, Incline Bench Press, Bent Over Row, Dead Lift; Day 2: Squat, Leg Curl, Crunch, and Shoulder Bridge; Day 3: Shoulder Press, Lateral Raise, Lying Extension, Biceps Curl) were performed in a single session. Rest between the sets of the exercise was set as 60–90 s. The repetition of resistance training was set as 15 reps a set at 70% 10RM intensity; 12 reps a set at 80% and 90% of 10RM intensity; and 10 reps a set at 100% of 10RM intensity.

The aerobic exercise was performed on motorized treadmills for at least 20 min and the intensity of exercise was corresponding to approximately 60–85% of age-predicted maximal heart rate [209–0.7 X age) [21] at the baseline. According to criteria from American College of Sports Medicine’s exercise testing guideline (classify the level of VO_2_max (mL/kg/min) by ‘very poor’, ‘poor’, ‘fair’, ‘good’, ‘excellent’, ‘superior’) [22], participants who were in the ‘very poor’, ‘poor’, and ‘fair’ levels, treadmill speed: 4–4–5-6-7-7-5-6-7-7-5-6-7-7-5-6-7-7-8-4 km/h (first minute → 20th minute) in every one minute was provided; and ‘good’, ‘excellent’, ‘superior’ levels, treadmill speed: 4–5–6-7-8-8-6-7-8-8-6-7-8-8-6-7-8-8-9-5 km/h in every one minute was provided.

### Analyses

All statistical analyses were performed using SPSS software, version 22.0 (IBM Corp., Armonk, NY: IBM Corp.). All analyses were conducted using ITT (Tables 2 and 3) and the results from PP were provided in the Additional file 1: Table S1 and Table S2. All values were presented as mean ± SD unless stated otherwise. To compare the baseline measurements with the post 8 week intervention measurements, paired Student’s t-test was performed. To compare the absolute change values, one-way ANOVA was used, and ‘Tukey’ post-hoc test was performed to find group differences. All results with *p-*values less than 0.05 were considered statistically significant. A conservative method of last observation carry forward was used to fill the missing measurements of participants who did to not finish the 8 week of intervention, or did not participate in the post-intervention measurements. Linear mixed model (LMM) analysis was conducted to find trending differences between groups. For LMM analysis, missing data was left as missing to get more accurate estimated values. The same analyses were conducted for PP approach, but imputations were not made for missing values.

## Results

### Participants and dropouts

**Fig. 1:**
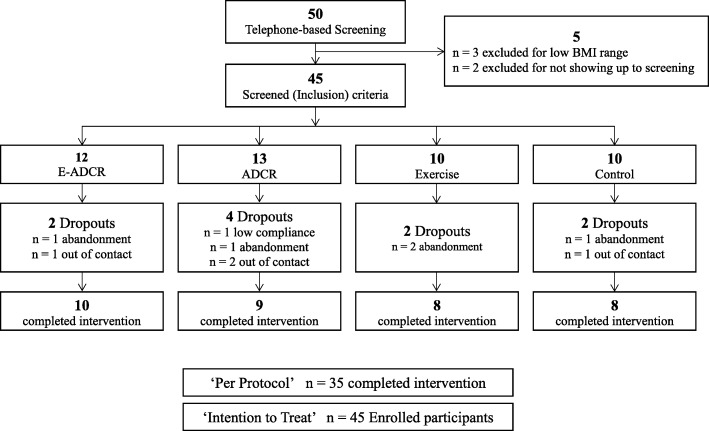
CONSORT Diagram (Study flow). Abbreviations, E-ADCR, Exercise plus alternate day calorie restriction, ADCR, Alternate day calorie restriction, BMI, Body mass index

Among the 50 participants, 3 participants who did not meet inclusion criteria (BMI ≥ 23.0 kg/m^2^), and 2 participants who did not attend the study orientation and the pre assessment, were excluded from the study. All participants were randomly allocated into 4 groups (E- ADCR = 12, ADCR = 13, exercise = 10, control = 10). Figure 1 shows flow of participants in study process and drop out. The data of the 45 participants (F = 26, M = 19) who participated in the orientation and pre assessment and commenced the 8 week intervention was used for ITT analyses (Tables 1, 2 and 3). Among the 45 participants, a total of 35 participants completed the trial (78%). Ten participants dropped out mainly due to ‘abandonment’ and ‘out of contact’ before the post assessment, and the remaining participants in each group were: E- ADCR = 10, ADCR = 9, exercise = 8, control = 8.

**Table 1:** Baseline characteristics of the participants

Variables	E-ADCR	ADCR	Exercise	Control	*p*-value
n	12	13	10	10	
Sex (F, M)	7, 5	10, 3	3, 7	6, 4	0.170
Age (years)	37.3 ± 7.3	32.9 ± 7.3	35.7 ± 7.9	40.6 ± 10.0	0.168
Height (cm)	163.2 ± 9.2	163.5 ± 7.1	170.8 ± 7.7	163.3 ± 6.9	0.086
Body weight (kg)	74.0 ± 13.7	74.1 ± 11.5	83.2 ± 16.7	70.8 ± 13.6	0.227
BMI (kg/m^2^)	27.5 ± 2.6	27.6 ± 2.8	28.3 ± 4.1	26.3 ± 3.0	0.548
Muscle mass (kg)	27.2 ± 7.4	26.6 ± 4.8	32.3 ± 7.2	26.5 ± 5.8	0.136
Fat mass (kg)	25.0 ± 4.6	25.9 ± 5.5	25.8 ± 7.2	22.9 ± 5.5	0.601
Percent body fat (%)	34.2 ± 6.1	34.9 ± 4.6	31.0 ± 5.0	32.2 ± 4.4	0.258
WC (cm)	90.5 ± 6.6	91.6 ± 7.9	95.5 ± 11.9	88.5 ± 7.5	0.328
SBP (mmHg)	120.8 ± 11.9	119.6 ± 16.7	125.3 ± 16.8	117.3 ± 14.4	0.683
DBP (mmHg)	80.3 ± 11.3	78.1 ± 14.0	83.5 ± 8.7	78.1 ± 10.2	0.668
HR (bpm)	78.4 ± 10.8	78.2 ± 9.4	74.2 ± 11.2	72.5 ± 12.6	0.511
Insulin (uIU/ml)	9.4 ± 3.5	9.3 ± 4.1	7.5 ± 3.6	8.5 ± 6.2	0.741
Glucose (mg/dl)	98.0 ± 17.0	90.0 ± 9.5	93.0 ± 8.0	87.0 ± 5.4	0.131
HOMA-IR	2.4 ± 1.4	2.1 ± 1.0	1.7 ± 0.9	1.8 ± 1.4	0.595
TG (mg/dl)	133.4 ± 47.1	108.8 ± 48.7	142.7 ± 78.3	109.5 ± 86.8	0.543
TC (mg/dl)	182.8 ± 26.0	189.8 ± 23.9	177.8 ± 33.4	178.5 ± 38.0	0.757
HDL-C (mg/dl)	48.0 ± 10.4	58.5 ± 15.0	44.8 ± 9.9	54.6 ± 11.8	0.041

Data are presented as mean ± SD

*E-ADCR* Exercise plus alternate day calorie restriction, *ADCR* Alternate day calorie restriction, *BMI* Body mass index, *WC* Waist circumference, *SBP* Systolic blood pressure, *DBP* Diastolic blood pressure, *HR* Heart rate, *HOMA-IR* Homeostasis model assessment-insulin resistance, *TG* Triglycerides, *TC* Total cholesterol, *HDL-C* High-density lipoprotein cholesterol

**Table 2:** Effect of intervention on anthropometric and body measurements (ITT)

Variables	Group	Baseline	Week 8	Change	*p*-value
Body weight (kg)	E-ADCR (*n* = 12)	74.0 ± 13.7	70.7 ± 14.2^a^	− 3.3 ± 2.4	0.064
	ADCR (*n* = 13)	74.1 ± 11.5	71.7 ± 11.0^b^	− 2.4 ± 3.1	
	Exercise (*n* = 10)	83.2 ± 16.7	81.8 ± 16.3	− 1.4 ± 2.1	
	Control (*n* = 10)	70.8 ± 13.6	70.2 ± 13.8	− 0.6 ± 1.3	
BMI (kg/m^2^)	E-ADCR (*n* = 12)	27.5 ± 2.6	26.3 ± 3.1^a^	− 1.3 ± 1.0^c^	0.036
	ADCR (*n* = 13)	27.6 ± 2.8	26.7 ± 2.5^b^	− 0.9 ± 1.3	
	Exercise (*n* = 10)	28.3 ± 4.1	27.9 ± 4.2^b^	− 0.5 ± 0.6	
	Control (*n* = 10)	26.3 ± 3.1	26.2 ± 3.0	−0.1 ± 0.5	
WC (cm)	E-ADCR (*n* = 12)	90.5 ± 6.6	86.4 ± 8.5^a^	−4.1 ± 3.9	0.267
	ADCR (*n* = 13)	91.6 ± 7.9	89.4 ± 8.3^b^	− 2.2 ± 3.6	
	Exercise (*n* = 10)	95.5 ± 11.9	92.6 ± 12.0^b^	− 2.9 ± 3.8	
	Control (*n* = 10)	88.5 ± 7.6	87.0 ± 8.9	− 1.5 ± 1.9	
Muscle mass (kg)	E-ADCR (*n* = 12)	27.2 ± 7.4	26.8 ± 7.5^b^	− 0.4 ± 0.5	0.696
	ADCR (*n* = 13)	26.6 ± 4.8	26.1 ± 4.6	− 0.5 ± 0.9	
	Exercise (*n* = 10)	32.3 ± 7.2	32.1 ± 6.8	− 0.1 ± 0.9	
	Control (n = 10)	26.5 ± 5.8	26.3 ± 6.1	− 0.2 ± 0.7	
Fat mass (kg)	E-ADCR (*n* = 12)	25.0 ± 4.6	22.3 ± 5.4^a^	− 2.7 ± 2.0^c^	0.036
	ADCR (n = 13)	26.0 ± 5.5	24.3 ± 6.4	− 1.6 ± 2.3	
	Exercise (*n* = 10)	25.8 ± 7.2	24.5 ± 7.1	− 1.2 ± 1.9	
	Control (*n* = 10)	22.9 ± 5.5	22.6 ± 5.7	−0.3 ± 1.3	
Percent body fat (%)	E-ADCR (*n* = 12)	34.2 ± 6.1	31.8 ± 6.8^a^	− 2.5 ± 2.2^c^	0.071
	ADCR (*n* = 13)	34.9 ± 4.6	33.6 ± 6.6^b^	−1.26 ± 2.4	
	Exercise (*n* = 10)	31.0 ± 5.0	29.9 ± 4.4	− 1.1 ± 1.8	
	Control (*n* = 10)	32.2 ± 4.4	32.2 ± 5.0	−0.1 ± 1.5	
SBP (mmHg)	E-ADCR (*n* = 12)	120.8 ± 11.9	117.5 ± 10.4	− 3.3 ± 8.3	0.389
	ADCR (*n* = 13)	119.6 ± 16.7	117.9 ± 17.1	−1.8 ± 5.8	
	Exercise (*n* = 10)	125.3 ± 16.8	119.5 ± 10.3	−5.8 ± 8.8	
	Control (n = 10)	117.3 ± 14.4	117.3 ± 14.6	0.0 ± 8.1	
DBP (mmHg)	E-ADCR (n = 12)	80.3 ± 11.3	77.9 ± 7.9	−2.3 ± 4.6	0.337
	ADCR (n = 13)	78.1 ± 14.0	77.0 ± 12.8	−1.4 ± 8.6	
	Exercise (n = 10)	83.5 ± 8.7	79.8 ± 10.5	−3.7 ± 8.3	
	Control (n = 10)	78.1 ± 10.2	79.9 ± 10.8	1.8 ± 5.0	

Data are presented as mean ± SD

*E-ADCR* Exercise plus alternate day calorie restriction, *ADCR* Alternate day calorie restriction, *BMI* Body mass index, *WC* Waist circumference, *SBP* Systolic blood pressure, *DBP* Diastolic blood pressure, *HR* Heart rate, *HOMA-IR* Homeostasis model assessment-insulin resistance, *TG* Triglycerides, *TC* Total cholesterol, *HDL-C* High-density lipoprotein cholesterol

^a^Significantly different between baseline and week 8, *p* < 0.01

^b^Significantly different between baseline and week 8, *p* < 0.05

^c^Significantly different with the control group, *p* < 0.05; *p* values were obtained by One-way ANOVA with Tukey post-hoc analysis

**Table 3:** Effect of intervention on blood lipids and insulin resistance (ITT)

Variables	Group	Baseline	Week 8	Change	*p*-value
Insulin (μIU/ml)	E-ADCR (n = 12)	9.4 ± 3.6	6.5 ± 3.0^b^	− 2.9 ± 4.1	0.205
	ADCR (n = 13)	9.3 ± 4.1	11.8 ± 12.9	2.6 ± 11.5	
	Exercise (n = 10)	7.5 ± 3.6	7.6 ± 3.5	0.1 ± 3.5	
	Control (n = 10)	8.5 ± 6.2	11.0 ± 7.7	2.6 ± 4.0	
Glucose (mg/dl)	E-ADCR (n = 12)	98.0 ± 17.0	87.08 ± 12.70^b^	−10.9 ± 16.9	0.110
	ADCR (n = 13)	90.0 ± 9.5	87.0 ± 8.1	−3.0 ± 9.4	
	Exercise (n = 10)	93.0 ± 8.0	92.3 ± 7.2	−0.7 ± 8.3	
	Control (n = 10)	87.0 ± 5.4	86.0 ± 5.7	− 1.0 ± 5.0	
HOMA-IR	E-ADCR (n = 12)	2.4 ± 1.4	1.4 ± 0.8^b^	− 0.9 ± 1.3	0.132
	ADCR (n = 13)	2.1 ± 1.0	2.7 ± 3.3	0.6 ± 2.8	
	Exercise (n = 10)	1.7 ± 0.9	1.8 ± 0.9	0.0 ± 0.8	
	Control (n = 10)	1.8 ± 1.4	2.4 ± 1.7	0.5 ± 0.9	
TG (mg/dl)	E-ADCR (n = 12)	133.4 ± 47.1	89.7 ± 33.7^a^	− 43.8 ± 41.9^c, d^	0.004
	ADCR (n = 13)	108.8 ± 48.7	120.8 ± 80.6	12.0 ± 56.2	
	Exercise (n = 10)	142.7 ± 78.3	106.9 ± 42.7	− 35.8 ± 55.8	
	Control (n = 10)	109.5 ± 86.8	130.7 ± 86.8	21.2 ± 31.0	
TC (mg/dl)	E-ADCR (n = 12)	182.8 ± 26.0	197.1 ± 28.6	14.3 ± 22.9	0.475
	ADCR (n = 13)	189.9 ± 23.91	193.9 ± 21.6	4.1 ± 19.6	
	Exercise (n = 10)	177.8 ± 33.4	191.9 ± 36.4	14.1 ± 25.3	
	Control (n = 10)	178.5 ± 38.0	194.8 ± 37.4^a^	16.3 ± 13.6	
HDL-C (mg/dl)	E-ADCR (n = 12)	48.0 ± 10.4	53.3 ± 10.5^b^	5.3 ± 8.1	0.622
	ADCR (n = 13)	58.5 ± 15.0	61.1 ± 15.4	2.6 ± 8.3	
	Exercise (n = 10)	44.8 ± 10.0	51.5 ± 8.6^b^	6.7 ± 8.5	
	Control (n = 10)	54.6 ± 11.8	59.4 ± 12.4^b^	4.9 ± 4.7	

Data are presented as mean ± SD

*E-ADCR* Exercise plus alternate day calorie restriction, *ADCR* Alternate day calorie restriction, *BMI* Body mass index, *WC* Waist circumference, *SBP* Systolic blood pressure, *DBP* Diastolic blood pressure, *HR* Heart rate, *HOMA-IR* Homeostasis model assessment-insulin resistance, *TG* Triglycerides, *TC* Total cholesterol, *HDL-C* High-density lipoprotein cholesterol

^a^Significantly different between baseline and week 8, *p* < 0.01

^b^Significantly different between baseline and week 8, *p* < 0.05

^c^Significantly different with the control group, *p* < 0.05

^d^Significantly different with the ADCR group, *p* < 0.05; *p* values were obtained by One-way ANOVA with Tukey post-hoc analysis

### Baseline characteristics

Baseline characteristics of all study participants are shown in Table 1. There is no significant difference between groups in age, height, weight, BMI, WC, level of blood pressure, and insulin resistance (Table 1).

### Body weight and body composition

Body weight of participants at baseline, post intervention, and values of change, are shown in Table 2. Participants in E- ADCR group lost a mean weight of 3.2 ± 2.4 kg (*p* < 0.01), and participants in ADCR group lost a mean weight of 2.4 ± 3.1 kg (*p* < 0.05) after the 8 weeks of intervention. BMI decreased by 1.3 ± 1.0 kg/m^2^ (*p* < 0.01) in E- ADCR group, 0.9 ± 1.3 kg/m^2^ (*p* < 0.05) in ADCR group, and 0.5 ± 0.6 kg/m^2^ (*p* < 0.05) in exercise group after the intervention. BMI also decreased by 0.1 ± 0.5 kg/m^2^ (*p* < 0.05) in control group. WC was significantly reduced by 4.1 ± 3.9 cm (*p* < 0.01) in E- ADCR group, 2.2 ± 3.6 cm (*p* < 0.05) in ADCR group, 2.9 ± 3.8 cm (*p* < 0.05) in exercise group after 8 weeks of intervention. Muscle mass in E-ADCR group decreased by 0.4 ± 0.5 kg (*p* < 0.05). Fat mass in E-ADCR group was reduced by 2.7 ± 2.0 kg (*p* < 0.01). Percent body fat in E-ADCR group also reduced by 2.5 ± 2.2% (*p* < 0.01), and 1.3 ± 2.4% (*p* < 0.05) in ADCR group. E-ADCR group showed a significant decrease in fat mass compared to control group (*p* < 0.05). LMM analysis showed significant trends between groups for weight (*p* = 0.035), BMI (*p* = 0.024) and fat mass (*p* = 0.028). When compared the change values, BMI (− 1.3 ± 1.0 vs. -0.1 ± 0.5), fat mass (− 2.7 ± 2.0 vs. -0.3 ± 1.3), and percent body fat (− 2.5 ± 2.2 vs. -0.1 ± 1.5) were significantly different between E-ADCR group and control group (*p* < 0.05).

### Blood lipids and insulin resistance

After the 8 weeks of intervention, significant decreases of 2.9 ± 4.1μIU/ml (*p* < 0.05) for insulin, 10.9 ± 16.9 mg/dl (*p* < 0.05) for glucose, 0.9 ± 1.3 (*p* < 0.05) for HOMA-IR, 43.8 ± 41.9 mg/dl (*p* < 0.01) for TG, and an increase of 5.3 ± 8.1 mg/dl (*p* < 0.05) for HDL-C were observed in E-ADCR group (Table 3). The level of HDL-C also increased by 6.7 ± 8.5 mg/dl (*p* < 0.05) in exercise group and 4.9 ± 4.7 mg/dl (*p* < 0.05) in control group. E-ADCR group showed significant decreases (*p* < 0.05) in TG compared to control group and ADCR group. LMM analysis showed significant trends between groups for TG (*p* < 0.01).

## Discussion

The purpose of this exploratory study was to investigate the effects of combining exercise and ADCR on body weight, body composition, blood lipids, and insulin resistance in overweight and obese adults. We hypothesized that E-ADCR groups would experience the largest reductions in cardio-metabolic risk factors, with the least decrease in lean muscle mass compared to other study groups. It is important for obese individuals to maintain or increase lean body mass during weight reductions because it directly affects one’s resting metabolic rate (RMR) and energy expenditure, which can contribute to substantial weight loss [23]. Previous literature has shown resistance exercise to attenuate the loss of lean muscle mass during caloric restriction [24]. Therefore, we expected that lean body mass would be maintained in the groups that participated in exercise, which includes resistance training exercise.

As we hypothesized, the largest decreases in body weight, BMI, WC, fat mass, and percent body fat were observed in E-ADCR group. Both diet intervention groups (E-ADCR and ADCR) experienced significant weight loss, and all intervention groups experienced a decrease in BMI and WC. Only participants in E-ADCR group experienced significant reductions in fat mass when compared to the control group, suggesting that combining ADCR with exercise results in more effective fat loss. The results of this study are consistent with a previous intervention study [16] that combined ADCR with endurance exercise, and showed that ADCR and exercise combined group experienced greater changes in body weight after 8 weeks of trial. However, contrary to prior literature [16, 25], our result indicated that muscle mass was reduced in E-ADCR group. A closer look at the results showed that the diet intervention group experienced greater losses of muscle mass, although both intervention and control groups lost muscle mass. Exercise group lost less muscle mass than control group, and E-ADCR group experienced less muscle mass loss than ADCR group. Therefore, the results of this study can be seen as being in line with previous literature, in that muscle mass decreased the least in groups that participated in exercise intervention.

For blood variables, the most beneficial changes can be seen in E-ADCR for fasting insulin, glucose, HOMA-IR, and TG levels. Previous literature has already demonstrated the correlation of glucose and insulin resistance with obesity [26], and its association with various coronary heart diseases [26–28], type 2 diabetes [29], and several types of cancers [30]. For this reason, treatment and management of glucose levels and insulin resistance in the overweight and obese population is crucial. The changes from baseline to post intervention measurements in this study reached statistical significance only in E-ADCR group, for fasting insulin, glucose, HOMA-IR, and TG levels. This suggests that caloric restriction in combination with exercise is the most beneficial method for lowering fasting insulin, glucose, HOMA-IR and TG. Our results show that after E-ADCR group, the exercise group showed the most beneficial reductions in fasting insulin and TG levels, followed by ADCR group. However, for fasting glucose, the most beneficial changes appear in E-ADCR group, followed by ADCR group, and then exercise group. This suggests that exercise may be more beneficial for reducing insulin and TG levels, while caloric restrictions may be more beneficial for reducing glucose levels. Similarly, the results for TC levels show an increase across all groups, with the least amount of increase in ADCR group, suggesting that caloric restrictions maybe more beneficial in controlling TC. The results for HDL-C show that the HDL-C levels increased across all groups, with the smallest increase in ADCR group. The largest increases can be seen in both exercise intervention groups (E-ADCR and exercise), which is in line with previous research that show that exercise increases HDL-C levels [31]; although control group showed a significant elevation of HDL-C after 8 weeks as well.

This study compares the effects of separate or combined ADCR and exercise on insulin resistance. A previous study has explored ADCR and endurance type exercise, but did not report any changes in insulin resistance, fasting glucose or insulin [16]. The current study show a significant decrease in the level of fasting insulin (− 2.9 ± 4.1μIU/ml), glucose (− 10.9 ± 16.9), and HOMA-IR (− 0.9 ± 1.3) in E-ADCR group only. The evidence from this exploratory study suggest that 8 weeks of intervention, combining ADCR with resistance training and aerobic type exercise, improves insulin resistance favorably. However, the study failed to compare the magnitude of effect between the interventions, because there were no significant differences of change value between the groups in insulin resistance. We may suggest that the ADCR plus combined exercise intervention reduced insulin resistance according to the result that only E-ADCR showed decrease in the value.

This exploratory study has several limitations. First, although a double blind design was initially implemented to elicit unpolluted results and to prevent any bias, the participants became aware of their respective groups during the diet and exercise education orientation. Second, the diet protocol of the study was not individualized and not supervised completely in all groups. Although metabolic rate of participants were different, the diet protocol (400–500 kcal of feeding in ‘calorie restriction day’) were identical for all participants in the ADCR groups. Therefore, outcome variables after 8 weeks might have some impacts, and also the increased HDL-C level in the control group after the intervention may happen. These points can cause a potential limitation. Third, this study did not control menstrual cycle of female participants. Since total cholesterol and HDL-C, or TG levels are affected by stages of menstrual cycle [32, 33], menstrual cycle should have been treated as a confounding variable. However, this study did not consider the possible impact of menstrual cycle on plasma lipids, this can be a limitation. Fourth, numbers of participants in each group were different because three participants were excluded after BMI measures, and two participants did not attend the first meeting (Fig. 1). Randomization was conducted after telephone screening according to the study exclusion and inclusion criteria. Fifth, physical activity levels of all the participants during the study intervention period were not reported and controlled. Each participant’s physical activity level may impact study outcomes because those are susceptible to physical activity. However, all the participants were asked to maintain pre-intervention physical activity level. Sixth, the study employed bio-impedance analyzer to measure body composition of study participants. The bio-impedance analyzer for body composition measures should be a limitation of this study, because a dual-energy absorptiometry technique is the gold standard for measuring body composition in clinical research study. Seventh, in regard to the exercise intervention, participants in both exercise groups underwent only one week of supervised exercise, and the remaining 7 weeks were unsupervised. Unsupervised exercise intervention and reporting the compliance by exercise log may overestimate or underestimate true value of exercise intervention [34]. Lastly, although study participants were stratified by sex and then randomly assigned into the four different study groups, unbalanced sex ratio between the groups may affect study results. Seeing as how all participants were unfamiliar with resistance exercise, it is reasonable to assume that a whole 8 weeks of supervised exercise training may show different result. Although this study has several limitations, this exploratory randomized controlled trial could identify the casual relationship between ADCR, exercise, and health-related outcomes in healthy overweight and obese adults.

## Conclusions

In summary, 8 weeks of the ADCR combined with exercise changed body composition, body weight, insulin resistance, TG, and HDL-C, although muscle mass was not maintained. Maintaining and increasing lean muscle mass is a key issue for the overweight population, especially during a calorie restriction diet; therefore, resistance training was included in this study. To our knowledge, this exploratory study is among the few to examine the combined effects of ADCR and exercise on body composition and cardio-metabolic risk factors in healthy overweight and obese adults. Future studies should include a larger population, and include more intensive, supervised resistance training in order to elucidate the effects of E-ADCR, ADCR and exercise. The results of this exploratory study show that ADCR in combination with exercise was the most effective at improving body weight, body composition, and blood lipids and insulin sensitivity in overweight and obese adults.

## Additional file

Additional file 1: **Table S1.** Effect of intervention on anthropometric and body measurements (PP). **Table S2.** Effect of intervention on blood lipids and insulin resistance (PP). (DOCX 26 kb)

## Abbreviations

ADCR: Alternate day calorie restriction
BMI: Body mass index
CHD: Coronary heart disease
E-ADCR: Exercise plus ADCR
HDL-C: Low density lipoprotein cholesterol
HOMA-IR: Homeostasis model assessment insulin resistance
ITT: Intention-to-treat
LDL-C: Low density lipoprotein cholesterol
LMM: Linear mixed model
RM: Repetition maximum
TC: Total cholesterol
TG: Triglyceride
WC: Waist circumference
