# Supplementary material for: Effects of alternate day calorie restriction and exercise on cardio-metabolic risk factors in overweight and obese adults: an exploratory randomized controlled study
Source: BMC Public Health. 2018 Sep 15;18:1124. doi: 10.1186/s12889-018-6009-1 (PMC6139127; doi:10.1186/s12889-018-6009-1)
Supplement: Supplementary file 1 — Table S1. Effect of intervention on anthropometric and body measurements (PP). Table S2. Effect of intervention on blood lipids and insulin resistance (PP). (DOCX 26 kb) [file 12889_2018_6009_MOESM1_ESM.docx]

| **Table S1.** Effect of intervention on anthropometric and body measurements (PP) | | | | | | | |
| --- | --- | --- | --- | --- | --- | --- | --- |
| **Variables** | **Group** | | **Baseline** | | **Week 8** | **Change** | ***p*-value** |
| Body weight (kg) | E-ADCR (n=10) | 75.0 ± 14.9 | | 71.1 ± 15.6^a^ | | -3.9 ± 2.1 | 0.028 |
|  | ADCR (n=9) | 75.9 ± 13.1 | | 72.5 ± 12.9^b^ | | -3.4 ± 3.3 |  |
|  | Exercise (n=8) | 79.5 ± 14.6 | | 77.8 ± 13.5 | | -1.7 ± 2.2 |  |
|  | Control (n=8) | 65.5 ± 9.0 | | 64.8 ± 8.9 | | -0.8 ± 1.5 |  |
| BMI (kg/m2) | E-ADCR (n=10) | 27.7 ± 2.8 | | 26.2 ± 3.4^a^ | | -1.5 ± 0.9^c^ | 0.016 |
|  | ADCR (n=9) | 28.0 ± 3.2 | | 26.8 ± 3.0^b^ | | -1.3 ± 1.4 |  |
|  | Exercise (n=8) | 27.2 ± 2.7 | | 26.7 ± 2.5^b^ | | -0.6 ± 0.6 |  |
|  | Control (n=8) | 25.3 ± 2.4 | | 25.2 ± 2.3 | | -0.1 ± 0.6 |  |
| WC (cm) | E-ADCR (n=9) | 90.7 ± 6.9 | | 85.7 ± 8.9^a^ | | -4.9 ± 3.7 | 0.216 |
|  | ADCR (n=7) | 91.8 ± 9.2 | | 88.7 ± 9.5^b^ | | -3.2 ± 4.0 |  |
|  | Exercise (n=7) | 92.1 ± 7.3 | | 88.5 ± 4.8^b^ | | -3.7 ± 3.9 |  |
|  | Control (n=8) | 86.1 ± 6.3 | | 84.2 ± 7.5^b^ | | -1.9 ± 2.0 |  |
| Muscle mass (kg) | E-ADCR (n=10) | 27.7 ± 7.7 | | 27.2 ± 7.8^b^ | | -0.5 ± 0.5 | 0.594 |
|  | ADCR (n=9) | 27.8 ± 5.3 | | 27.1 ± 5.1 | | -0.7 ± 1.0 |  |
|  | Exercise (n=8) | 31.2 ± 7.6 | | 31.0 ± 7.0 | | -0.2 ± 1.0 |  |
|  | Control (n=8) | 24.2 ± 3.6 | | 23.9 ± 3.9 | | -0.3 ± 0.8 |  |
| Fat mass (kg) | E-ADCR (n=10) | 25.3 ± 3.7 | | 22.0 ± 4.9^a^ | | -3.3 ± 1.7^c^ | 0.021 |
|  | ADCR (n=9) | 25.9 ± 6.6 | | 23.6 ± 7.6^b^ | | -2.3 ± 2.4 |  |
|  | Exercise (n=8) | 23.9 ± 4.5 | | 22.4 ± 3.3 | | -1.5 ± 2.0 |  |
|  | Control (n=8) | 21.4 ± 5.0 | | 21.0 ± 5.2 | | -0.3 ± 1.4 |  |
| Percent body fat (%) | E-ADCR (n=10) | 34.2 ± 4.5 | | 31.2 ± 5.4^a^ | | -2.9 ± 2.0 | 0.065 |
|  | ADCR (n=9) | 33.9 ± 5.2 | | 32.1 ± 7.4 | | -1.8 ± 2.7 |  |
|  | Exercise (n=8) | 30.4 ± 5.0 | | 29.1 ± 3.9 | | -1.3 ± 1.9 |  |
|  | Control (n=8) | 32.4 ± 4.9 | | 33.0 ± 5.7 | | -0.1 ± 1.7 |  |
| SBP (mmHg) | E-ADCR (n=10) | 122.1 ± 12.7 | | 118.1 ± 11.3 | | -4.0 ± 9.0 | 0.414 |
|  | ADCR (n=9) | 119.2 ± 8.5 | | 116.7 ± 9.2 | | -2.6 ± 7.0 |  |
|  | Exercise (n=8) | 125.3 ± 18.9 | | 118.0 ± 10.9 | | -7.3 ± 9.3 |  |
|  | Control (n=8) | 115.5 ± 13.4 | | 115.5 ± 13.6 | | 0.0 ± 9.2 |  |
| DBP (mmHg) | E-ADCR (n=10) | 81.2 ± 12.3 | | 78.4 ± 8.6 | | -2.8 ± 5.0 | 0.354 |
|  | ADCR (n=9) | 76.8 ± 11.9 | | 74.8 ± 9.3 | | -2.0 ± 10.4 |  |
|  | Exercise (n=8) | 81.9 ± 9.0 | | 77.3 ± 10.1 | | -4.6 ± 9.1 |  |
|  | Control (n=10) | 76.6 ± 7.0 | | 78.9 ± 8.3 | | 2.3 ± 5.5 |  |

Data are presented as mean ± SD. E-ADCR, Exercise plus alternate day calorie restriction; ADCR, Alternate day calorie restriction; BMI, Body mass index; WC, Waist circumference; SBP, Systolic blood pressure; DBP, Diastolic blood pressure; HR, Heart rate; HOMA-IR, Homeostasis model assessment-insulin resistance; TG, Triglycerides; TC; Total cholesterol; HDL-C, High-density lipoprotein cholesterol. ^a^ Significantly different between baseline and week 8, *p* < 0.01; ^b^ Significantly different between baseline and week 8, *p* < 0.05; ^c^ Significantly different with the control group, *p* < 0.05; *p* values were obtained by One-way ANOVA with Tukey post-hoc analysis.

| **Table S2.** Effect of intervention on blood lipids and insulin resistance (PP) | | | | | |
| --- | --- | --- | --- | --- | --- |
| **Variables** | **Group** | **Baseline** | **Week 8** | **Change** | ***p*-value** |
| Insulin (μIU/ml) | E-ADCR (n=10) | 10.0 ± 3.5 | 6.5 ± 3.2^b^ | -3.5 ± 4.3 | 0.204 |
|  | ADCR (n=9) | 9.5 ± 4.9 | 13.2 ± 15.6 | 3.7 ± 13.9 |  |
|  | Exercise (n=8) | 6.4 ± 2.3 | 6.5 ± 2.2 | 0.2 ± 3.9 |  |
|  | Control (n=8) | 6.1 ± 4.0 | 9.3 ± 7.6 | 3.2 ± 4.3 |  |
| Glucose (mg/dl) | E-ADCR (n=10) | 99.4 ± 18.3 | 86.3 ± 13.7^b^ | -13.1 ± 17.8 | 0.134 |
|  | ADCR (n=9) | 94.0 ± 8.4 | 89.7 ± 8.2 | -4.3 ± 11.2 |  |
|  | Exercise (n=8) | 91.5 ± 8.3 | 90.6 ± 7.0 | -0.9 ± 9.4 |  |
|  | Control (n=8) | 86.4 ± 4.8 | 85.1 ± 5.1 | -1.3 ± 5.6 |  |
| HOMA-IR | E-ADCR (n=10) | 2.6 ± 1.4 | 1.4 ± 0.9^b^ | -1.1 ± 1.4 | 0.137 |
|  | ADCR (n=9) | 2.2 ± 1.1 | 3.1 ± 4.0 | 0.9 ± 3.4 |  |
|  | Exercise (n=8) | 1.4 ± 0.5 | 1.5 ± 0.5 | 0.0 ± 0.9 |  |
|  | Control (n=8) | 1.3 ± 0.8 | 2.0 ± 1.6 | 0.7 ± 0.9 |  |
| TG (mg/dl) | E-ADCR (n=10) | 137.8 ± 50.3 | 85.3 ± 34.6^a^ | -52.5 ± 40.5^c^ | 0.002 |
|  | ADCR (n=7) | 91.4 ± 42.4 | 85.0 ± 47.4 | -6.4 ± 47.5 |  |
|  | Exercise (n=7) | 115.3 ± 50.4 | 91.6 ± 34.3 | -23.7 ± 36.9 |  |
|  | Control (n=8) | 78.8 ± 23.3 | 105.3 ± 47.3 | 26.5 ± 32.8 |  |
| TC (mg/dl) | E-ADCR (n=10) | 182.7 ± 25.1 | 199.9 ± 27.3 | 17.2 ± 24.2 | 0.562 |
|  | ADCR (n=9) | 190.6 ± 22.7 | 196.4 ± 19.0 | 5.9 ± 23.8 |  |
|  | Exercise (n=8) | 175.1 ± 35.9 | 192.8 ± 39.9 | 17.6 ± 27.5 |  |
|  | Control (n=8) | 173.5 ± 33.9 | 193.9 ± 35.1^a^ | 20.4 ± 12.0 |  |
| HDL-C (mg/dl) | E-ADCR (n=10) | 46.7 ± 8.4 | 53.1 ± 9.2^b^ | 6.4 ± 8.5 | 0.713 |
|  | ADCR (n=9) | 55.1 ± 15.0 | 58.8 ± 16.3 | 3.7 ± 10.0 |  |
|  | Exercise (n=8) | 44.3 ± 10.0 | 52.7 ± 7.8^b^ | 8.4 ± 8.8 |  |
|  | Control (n=8) | 56.7 ± 12.4 | 62.7 ± 11.7^a^ | 6.1 ± 4.5 |  |

Data are presented as mean ± SD. E-ADCR, Exercise plus alternate day calorie restriction; ADCR, Alternate day calorie restriction; BMI, Body mass index; WC, Waist circumference; SBP, Systolic blood pressure; DBP, Diastolic blood pressure; HR, Heart rate; HOMA-IR, Homeostasis model assessment-insulin resistance; TG, Triglycerides; TC; Total cholesterol; HDL-C, High-density lipoprotein cholesterol. ^a^ Significantly different between baseline and week 8, *p* < 0.01; ^b^ Significantly different between baseline and week 8, *p* < 0.05; ^c^ Significantly different with the control group, p < 0.05; *p* values were obtained by One-way ANOVA with Tukey post-hoc analysis
